# Hepatoprotective Effect of Steroidal Glycosides From *Dioscorea villosa* on Hydrogen Peroxide-Induced Hepatotoxicity in HepG2 Cells

**DOI:** 10.3389/fphar.2018.00797

**Published:** 2018-07-23

**Authors:** Maqsood A. Siddiqui, Zulfiqar Ali, Amar G. Chittiboyina, Ikhlas A. Khan

**Affiliations:** ^1^Zoology Department, College of Science, King Saud University, Riyadh, Saudi Arabia; ^2^Al-Jeraisy Chair for DNA Research, Zoology Department, College of Science, King Saud University, Riyadh, Saudi Arabia; ^3^National Center for Natural Products Research, School of Pharmacy, The University of Mississippi, Oxford, MS, United States; ^4^Department of BioMolecular Sciences, School of Pharmacy, The University of Mississippi, Oxford, MS, United States

**Keywords:** *Dioscorea villosa*, Dioscoreaceae, steroidal glycosides, H_2_O_2_, cytotoxicity, ROS generation

## Abstract

*Dioscorea villosa*, commonly known as “Wild Yam” and native to North America, is well documented for its pharmacological properties due to the presence of steroidal glycosides. However, the hepatoprotective potential of these compounds has not been studied so far. The present investigation was aimed to study the hepatoprotective effect of the steroidal glycosides from *D. villosa* against H_2_O_2_, a known hepatotoxin, in human liver cell line (HepG2). Cytotoxicity assessment was carried out in cells exposed to various concentrations (10–50 μM) of compounds for 24 h using MTT assay and morphological changes. All tested compounds were known and among them, spirostans (zingiberensis saponin I, dioscin, deltonin and progenin III) were found to be cytotoxic whereas, furostans (huangjiangsu A, pseudoprotodioscin, methyl protobioside, protodioscin, and protodeltonin) were non-cytotoxic. Further, HepG2 cells were pretreated with biologically safe concentrations (10, 30, and 50 μM) of non-cytotoxic compounds and then cytotoxic (0.25 mM) concentration of H_2_O_2_. After 24 h, cell viability was assessed by MTT and NRU assays, while morphological changes were observed under the microscope. The results showed that treatment of HepG2 cells with compounds prior to H_2_O_2_ exposure effectively increased cell viability in a concentration-dependent manner. Furthermore, huangjiangsu A, pseudoprotodioscin, methyl protobioside, protodioscin, and protodeltonin at 50 μM increased GSH level and decreased intracellular ROS generation against H_2_O_2_-induced damages. The results from this study revealed that compounds isolated from *D. villosa* have hepatoprotective potential against H_2_O_2_-induced cytotoxicity and ROS generation and could be promising as potential therapeutic agents for liver diseases.

## Introduction

The liver is the largest glandular organ, and plays a key role in the regulation of various physiological processes in the human body (Bechmann et al., 2012). It is the most important site of intermediary metabolism responsible for the detoxification and excretion of various substances like xenobiotics by altering and expelling toxins and wastes (Chaudhari and Mahajan, 2016). Owing to continuous exposure to the toxicant via the portal blood circulation, the liver is highly susceptible to damage by xenobiotics, drugs, and hazardous substances (Sharma et al., 2011). Due to handling the metabolism of various proteins, lipids, carbohydrates, secretion of bile, and storage of vitamins, it is considered as one of the most vital organs (Bechmann et al., 2012). The liver diseases are a global health problem therefore, the maintenance of healthy liver is imperative for human health (Haidry and Malik, 2014). A variety of chemicals, such as tert-butyl hydroperoxide (t-BHP), D-galactosamine/lipopolysaccharide, carbon tetrachloride (CCl_4_), acetaminophen, alcohol, and hydrogen peroxide can cause potential damage to the liver cells leading to progressive dysfunction (Simeonova et al., 2014). Oxidative stress is a redox imbalance between pro-oxidant, i.e., the production of reactive oxygen species (ROS) and antioxidant defense counteracting the reactive intermediates and initiating the cellular damage (Videla, 2009). Oxidative stress is also known to be involved in the mechanism of hepatotoxicity and pathogenesis (Li et al., 2015). Overproduction of ROS results in oxidative stress, a deleterious process that can be an important mediator of damage to cell structures, lipids, membranes, proteins, and DNA (Ray et al., 2012). Hydrogen peroxide (H_2_O_2_) is well known to increase the levels of intracellular ROS generation and causes cellular oxidative damage (Cheong et al., 2016). Several evidences also showed that H_2_O_2_-induced cytotoxicity in human liver cells (Al-Sheddi et al., 2016; Gao et al., 2016) and has been linked with various alterations including anti- and pro-apoptosis proteins and caspases (Cheong et al., 2016). Therefore, H_2_O_2_ was chosen in this study as an inducer of oxidative stress and cytotoxicity in the human liver cell line (HepG2). Despite the recent therapeutic advances and significant development of modern medicine, hepatic diseases remain a health problem worldwide (Hong et al., 2015). Thus, the search for new therapeutic agents to treat liver diseases is still in demand. Many synthetic drugs have been demonstrated to be strong radical scavengers, but they are also carcinogenic and cause liver damage (Cheong et al., 2016). Due to the fact that the available drugs can cause damage to healthy hepatic cells, the new researches have been focused to protect hepatic diseases and reduce adverse side effects by using natural compounds isolated from the plants. A literature survey revealed that many plant extracts and their constituents have constantly shown hepatoprotective effects (Jiménez-Arellanes et al., 2016). It has also been reported that pure compounds and extracts from plants possess good antioxidant activity against chemically induced liver damage (Pereira et al., 2016).

*Dioscorea villosa* L. (Family: Dioscoreaceae), a tuber vegetable commonly known as “Wild Yam,” is native to North America and has been recommended for menopausal symptoms and menstrual complaints (Komesaroff et al., 2001). *Dioscorea* plants are rich in steroidal saponins and sapogenins. Over 50 steroid saponins of furostane-, spirostane-, and pregnane-type skeletons have been isolated and characterized from various *Dioscorea* species, which have been reported to be the major physiologically active constituents in yams (Hayes et al., 2007; Ali et al., 2013a,b). Among the isolated constituents of *D. villosa*, the cytotoxic activities of diosgenin and dioscin have been studied against a number of cancer cell lines (Kim et al., 2014; Aumsuwan et al., 2016). Hypocholesterolemic and hypoglycemic effects of dioscin have also been reported (McAnuff et al., 2005) and no subchronic toxicity of dioscin was observed *in vivo* with a high dose for 3 months (Xu et al., 2012). A number of preclinical and mechanistic studies have been reported supporting the antitumor potential of dioscin (Aumsuwan et al., 2016). The epigenetic mechanism of the anticancer effect of wild yam root extract (Aumsuwan et al., 2015) and a detailed study on anticancer potential of dioscin on cell viability, invasion, migration, and wound healing in an invasive breast cancer cell line has been reported (Aumsuwan et al., 2016). These studies demonstrated that constituents of *D. villosa* possess pharmacological potential especially antitumor effects. To the best of our knowledge, there are no reports available on their hepatoprotective effect. Therefore, this study was aimed to investigate the hepatoprotective effect of the constituents of *D. villosa* against H_2_O_2_-induced cytotoxicity and oxidative stress in HepG2 cell line. HepG2 cell line is well reported to be involved in the metabolism (Kim et al., 2011) and have been proven to be a good model system for cytotoxicity assessment and cyto-protective potential of various compounds against the toxic substances (González et al., 2017).

## Materials and Methods

### Chemicals and Consumables

Dulbecco’s modified Eagle medium (DMEM) culture medium, trypsin, fetal bovine serum (FBS), and antibiotics/antimycotic solution were procured from Invitrogen, Life Technologies, Grand Island, NY, United States. Plastic wares and other consumables used in the study were purchased from Nunc, Roskilde, Denmark. H_2_O_2_ and all specified chemicals and reagents were purchased from Sigma Chemical Company Pvt., Ltd., St. Louis, MO, United States.

### Plant Material

Root powder of *Dioscorea villosa* L. was purchased from Starwest Botanicals, United States. A sample specimen of the purchased material (No. 9412) was deposited at the National Center for Natural Products Research, The University of Mississippi.

### Extraction and Isolation

Root powder (0.9 kg) was extracted with MeOH (3.0 L × 4 × 24 h) at room temperature. Following the removal of the solvent, a gummy residue (MeOH extract, 75 g) was obtained. An aliquot (55 g) was subjected to size exclusion chromatography using Sephadex LH-20 (17 × 10 cm) and eluted with MeOH to furnish 2 parts, A (7.0 L, 45.2 g) and B (5.0 L, 9.4 g). Part A (30 g) was separated by normal phase column chromatography (NPCC) using silica gel (30 × 10 cm) into 12 fractions (A1–A12) with lower layer of CHCl_3_-MeOH-H_2_O (13:7:2) [A1 (0.5 L, 782 mg), A2 (1.0 L, 730 mg), A3 (0.5 L, 304 mg), A4 (0.5 L, 767 mg), A5 (0.5 L, 490 mg), A6 (0.5 L, 1.8 g), A7 (1.0 L, 657 mg), A8 (1.0 L, 2.8 g), A9 (1.0 L, 1.9 g), A10 (1.5 L, 4.4 g), A11 (1.5 L, 4.5 g), and A12 (1.5 L, 5.9 g)]. Fractions A3 and A4 were individually subjected to NPCC [silica gel (87 × 1.3 cm), EtOAc-CHCl_3_-MeOH-H_2_O (10:6:4:1), 1.0 L] to yield progenin III (375 mg) and dioscin (126 mg). Deltonin (785 mg) and zingiberensis saponin I (90 mg) were obtained from fractions A6 and A8, respectively, as MeOH insoluble materials. Methyl protobioside (325 mg) was purified from the MeOH soluble part of fraction A8 by NPCC [silica gel (87 × 2.5 cm), CHCl_3_-MeOH-H_2_O (32:8:1), 4.5 L] and RPC [RP-18 silica gel (37 × 2.5 cm), acetone-H_2_O (1:1), 0.5 L]. Fraction A9 was resolved by NPCC [silica gel (37 × 2.5 cm), lower layer of CHCl_3_-MeOH-H_2_O (13:7:2), 2.7 L] into three subfractions (A9a–A9c). Pseudoprotodioscin (45 mg) and protodioscin (212 mg) were purified from subfraction A9c (665 mg) by RPC [RP-18 silica gel (45 × 2.5 cm), acetone-H_2_O (2:3), 0.7 L]. Huangjiangsu A (646 mg) and protodeltonin (1.3 g) were purified from fraction A11 by RPC [RP-18 silica gel (50 × 3.7 cm), acetone-H_2_O (2:3), 2.5 L].

### Cell Culture

HepG2 cell line was purchased from ATCC and was cultured in DMEM, supplemented with 10% FBS, 0.2% sodium bicarbonate and antibiotic/antimycotic solution (1 mL/100 mL of medium). Cells were grown in a CO_2_ incubator at 37°C. Prior to the experiments, viability of cells was assessed using the previously described protocol (Siddiqui et al., 2008). Batches showing more than 98% cell viability and passage number of cells between 22 and 24 were used in this study.

### Experimental Design

For cytotoxicity assessments, HepG2 cells were exposed to various concentrations (10–50 μM) of the *D. villosa* compounds and H_2_O_2_ (0.025–2 mM) for 24 h. To study the cytoprotective potential, HepG2 cells were pretreated with biologically safe concentrations (10, 30, and 50 μM) of non-cytotoxic *D. villosa* compounds (huangjiangsu A, pseudoprotodioscin, methyl protobioside, protodioscin, and protodeltonin) and then cytotoxic concentrations of H_2_O_2_ (0.25 mM) were added in the medium containing compounds for 24 h. Further, for protective potential of compounds on GSH level and intracellular ROS generation against H_2_O_2_, cells were pre-exposed to 50 μM of the *D. villosa* compounds for 24 h then, H_2_O_2_ (0.25 mM) for 24 h.

### Preparation of Stock Solutions of Compounds

The stock solutions of all the compounds (25 mM) were prepared in dimethylsulfoxide (DMSO) and diluted in culture medium to reach the desired concentrations. The final DMSO concentration used for the experiments was not more than 0.2% and this was used as control.

### Cytotoxicity Assessment by MTT Assay

The MTT assay was performed for cytotoxicity assessments following the method described (Siddiqui et al., 2008). In brief, HepG2 cells were seeded in 96-well plates at a density of 1 × 10^4^ cells per well and allowed to grow overnight. Then, cells were exposed to various concentrations of compounds of *D. villosa* for 24 h. After the exposure, 50 μg MTT was added in each well and plates were incubated further for 4 h. Then, the reaction mixture was carefully taken out and 200 μL of DMSO was added in each well and mixed gently by pipetting up and down. The absorbance of plates was measured at 550 nm.

### Cytotoxicity Assessment by Neutral Red Uptake (NRU) Assay

Neutral red uptake (NRU) assay was performed according to the method described (Siddiqui et al., 2008). In brief, cells were exposed to various concentrations of compounds of *D. villosa* for 24 h. After the exposure, the test solution was aspirated and cells were washed twice with PBS and incubated further in medium containing 50 μg/mL neutral red for 3 h. Then, the medium was washed with a solution of CaCl_2_ (1%) and formaldehyde (0.5%). Following 20 min incubation at 37°C in a mixture of acetic acid (1%) and ethanol (50%), the plates were read at 550 nm.

### Morphological Changes by Phase Contrast Microscope

The alterations in the morphology was observed under the inverted microscope to determine the changes induced by compounds of *D. villosa* in HepG2 cells exposed to 10–50 μM for 24 h. The cell images were grabbed using phase contrast inverted microscope (Olympus) at 20× magnification.

### Glutathione (GSH) Level

The level of glutathione in HepG2 cells was measured following the method (Chandra et al., 2002). In brief, HepG2 cells were exposed to 50 μM of the compounds of *D. villosa* for 24 h and then H_2_O_2_ (0.25 mM) for 24 h. After respective exposure, cells were centrifuged and cellular protein was precipitated with 1 mL TCA (10%) on ice for 1 h. Supernatant was taken by centrifugation at 956 × *g* for 10 min. Then, 2 mL of 0.4 M Tris buffer (pH 8.9) containing 0.02 M EDTA and 0.01 M 5,5′-dithionitrobenzoic acid (DTNB) were added in the supernatant. The absorbance of the developed yellow color was read at 412 nm after incubating for 10 min at 37°C.

### Reactive Oxygen Species (ROS) Generation

The intracellular ROS generation was measured using 2,7-dichlorodihydrofluorescein diacetate (DCFH-DA) dye (Siddiqui et al., 2013). HepG2 cells were exposed to 50 μM of the compounds for 24 h and then H_2_O_2_ (0.25 mM) was added in the medium containing compounds for 24 h. After the respective exposure, HepG2 cells were washed with PBS and incubated with DCFH-DA (5 μM) at 37°C for 1 h in dark. The fluorescence of cells was analyzed using a fluorescence microscope (Olympus).

### Statistical Analysis

Data were statistically analyzed by ANOVA and results are presented as mean ± standard deviation (SD) of three separate experiments (*N* = 6). The treated and control groups were compared using the *post hoc* Dunnett’s test using statistical analysis software GraphPad Prism and considered a significant level of *p* < 0.05.

## Results and Discussion

### Compounds

**FIGURE 1:**
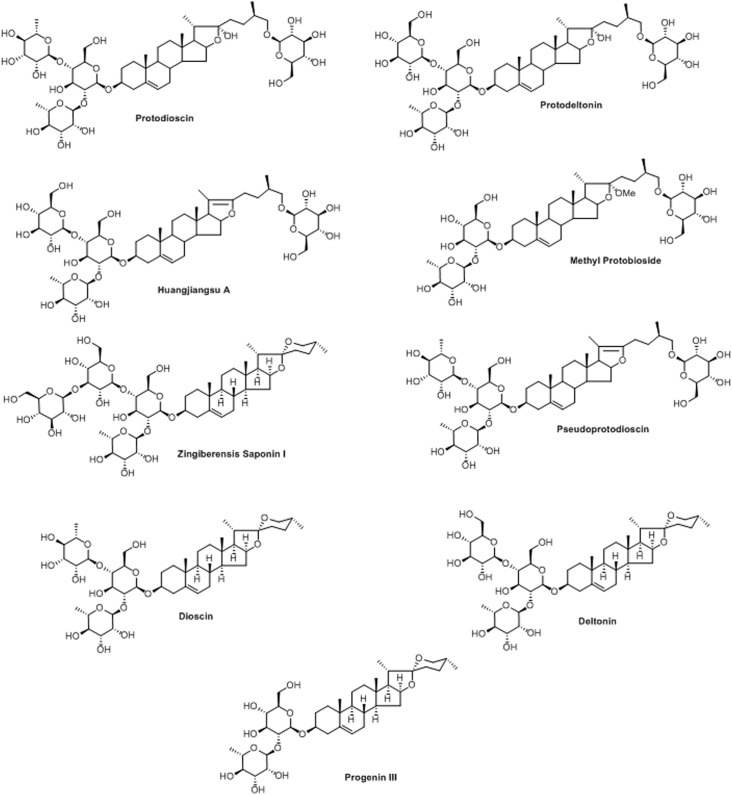
Structures of isolated compounds from *Dioscorea villosa*.

The molecular formula, C_51_H_82_O_21_, of pseudoprotodioscin was determined from an [M+Na]^+^ ion peak in the HRESIMS at *m/z* 1053.5347 and 51 carbon resonances in its ^13^C NMR spectrum attributable to furostan steroid and four sugar units. Its structure was elucidated by analyzing 1D and 2D NMR spectral data which were comparable with those of published in the literature (Yoshikawa et al., 2007). An [M+H]^+^ ion peak at *m/z* 1047.5482 in the HRESIMS and 51 carbon resonances (ascribable to furostan steroid and four sugar units) in the ^13^C NMR spectrum of huangjiangsu A, established its molecular formula as C_51_H_82_O_22_. Its structure was elucidated by analyzing the NMR data, which were analogous to those of published in the literature (Ali et al., 2013c). Methyl protobioside (Silva et al., 1998; Shen et al., 2002), protodioscin (Shao et al., 1997), and protodeltonin (Munafo et al., 2010) were also identified as furostan steroid glycosides by comparing their NMR data with those of reported. Methyl protobioside, protodioscin, and protodeltonin showed [M+Na]^+^ ion peaks in their HRESI mass spectra at *m/z* 939.5056 (corresponded to molecular formula of C_46_H_76_O_18_), *m/z* 1071.5453 (corresponded to molecular formula of C_51_H_84_O_22_), and *m/z* 1087.5383 (corresponded to molecular formula of C_51_H_84_O_23_), respectively. Zingiberensis saponin I, progenin III, dioscin, and deltonin were identified as spirostan steroidal glycosides by analyzing their 1D and 2D NMR spectroscopic and HRESIMS spectrometric data as well as by comparing their NMR data with those of published in the literature (Hayes et al., 2007). The HRESI mass spectra of zingiberensis saponin I, progenin III, dioscin, and deltonin displayed [M+Na]^+^ ion peaks at *m/z* 1069.5209 (C_51_H_82_O_22_+Na), *m/z* 745.4219 (C_39_H_62_O_12_+Na), *m/z* 891.4803 (C_45_H_72_O_16_+Na), and *m/z* 907.4587 (C_45_H_72_O_17_+Na), respectively. The structures of the isolated compounds are given in **Figure 1** and their purities were found to be over 95%, except for protodioscin (90% pure).

### Cytotoxicity Assessment of Compounds and H_2_O_2_

**FIGURE 2:**
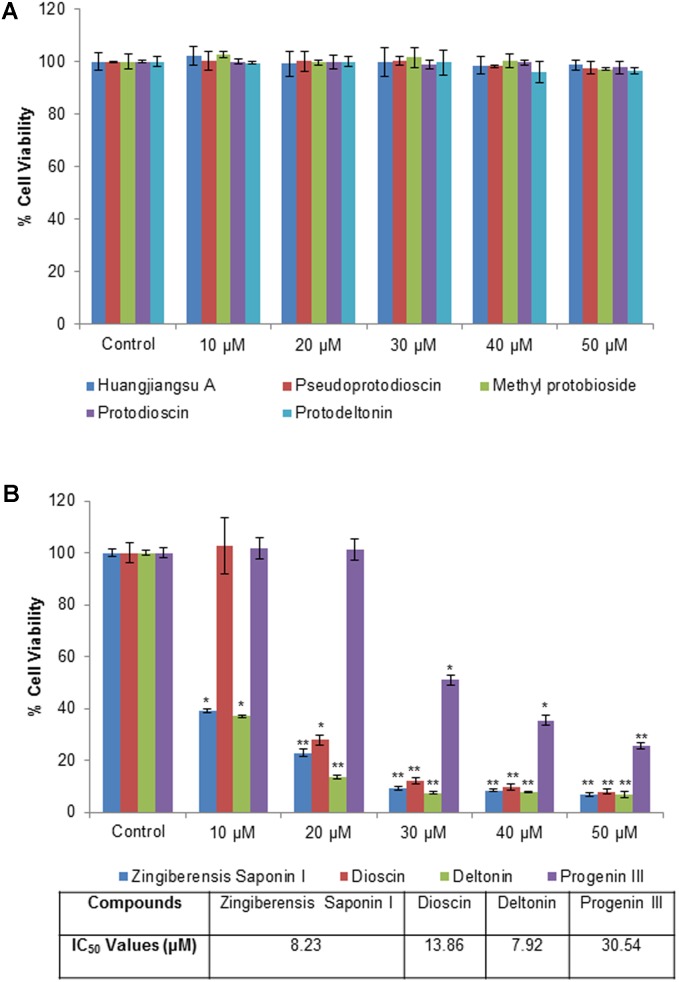
Cytotoxicity assessment by MTT assay in HepG2 cells following the exposure of different concentrations of compounds isolated from *Dioscorea villosa*. All values are given as mean ± SD. **(A)** Non-cytotoxic compounds and **(B)** Cytotoxic compounds. ^∗^*p* < 0.05, ^∗∗^*p* < 0.01 versus control.

**FIGURE 3:**
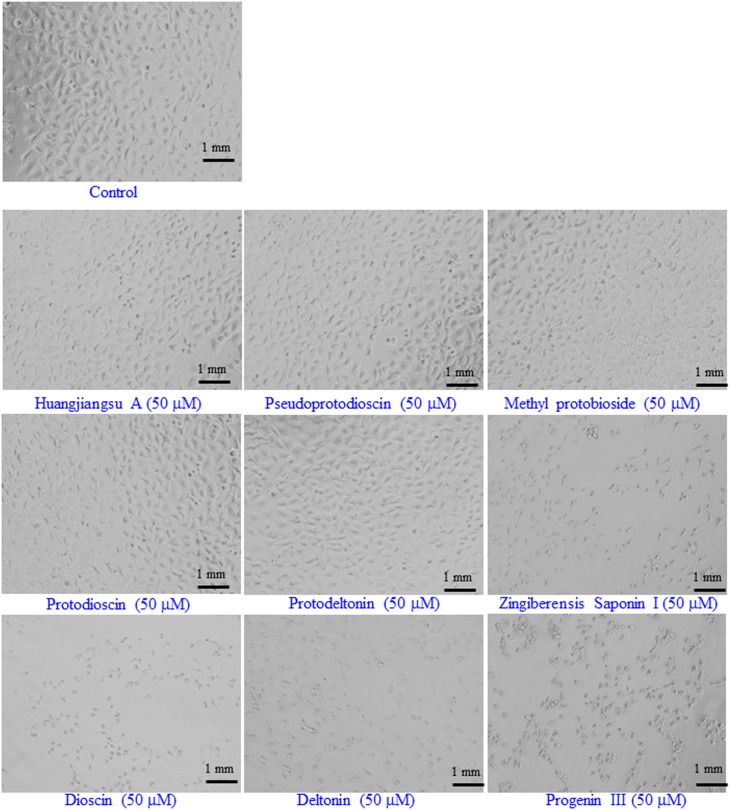
Representative images of the morphological changes in HepG2 cells after the exposure of the compounds isolated from *Dioscorea villosa*. All the images were acquired using a phase contrast inverted microscope at 20× magnification. Each scale bar = 1 mm.

**FIGURE 4:**
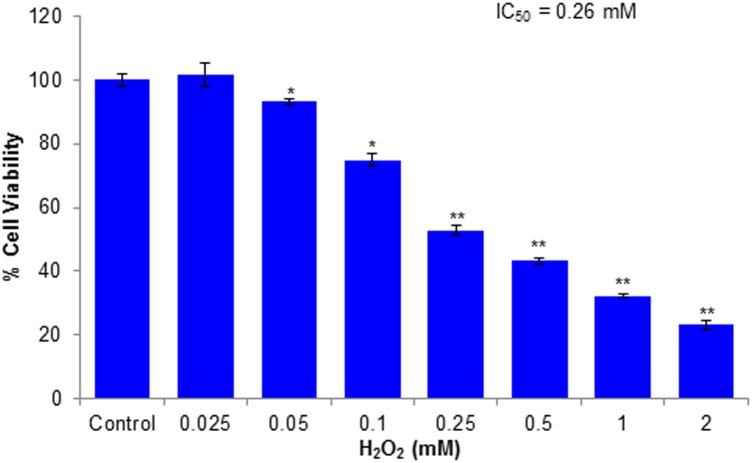
Cytotoxicity assessments by MTT assay in HepG2 cells following the exposure of various concentrations of H_2_O_2_ for 24 h. All values are given as mean ± SD. ^∗^*p* < 0.05, ^∗∗^*p* < 0.01 versus control.

The cytotoxic effects of compounds have been assessed by MTT assay and morphological changes. The results (**Figures 2**, **3**) showed that huangjiangsu A, pseudoprotodioscin, methyl protobioside, protodioscin, and protodeltonin (furostane-type steroidal glycosides) did not cause any effects on cell viability of HepG2 cells (**Figure 2A**) at 10–50 μM concentrations. However, some compounds, i.e., zingiberensis saponin I (furostane-type steroidal glycoside), dioscin, deltonin, and progenin III (spirostane-type steroidal glycosides) were found to be cytotoxic (**Figure 2B**). Moreover, the cytotoxic compounds induced cell damage was complemented by changes in cell morphology as observed in the loss of the characteristics in round form; however, no change was observed in non-cytotoxic compounds at highest concentration, i.e., 50 μM (**Figure 3**). Further, the non-cytotoxic compounds were selected to study the hepatoprotective effect against H_2_O_2_-induced cytotoxicity in HepG2 cells. As shown in **Figure 4**, H_2_O_2_ ranging from 0.05 to 2 mM induced cell death in a concentration-dependent manner. Based on the cytotoxicity data, the 0.25 mM concentration of H_2_O_2_-induced cytotoxicity in a moderate manner, therefore, this concentration (0.25 mM) of H_2_O_2_ was used for all further experiments. The results of this study are consistent with the previous reports of concentration-dependent cytotoxicity of H_2_O_2_ in HepG2 (Al-Sheddi et al., 2016) and other cells (Sattayasai et al., 2013). These studies also demonstrated that H_2_O_2_ could be used as a substance to induce oxidative stress mediated cytotoxicity in cell system.

### Cytoprotective Potential of Compounds Against H_2_O_2_-Induced Cytotoxicity

**FIGURE 5:**
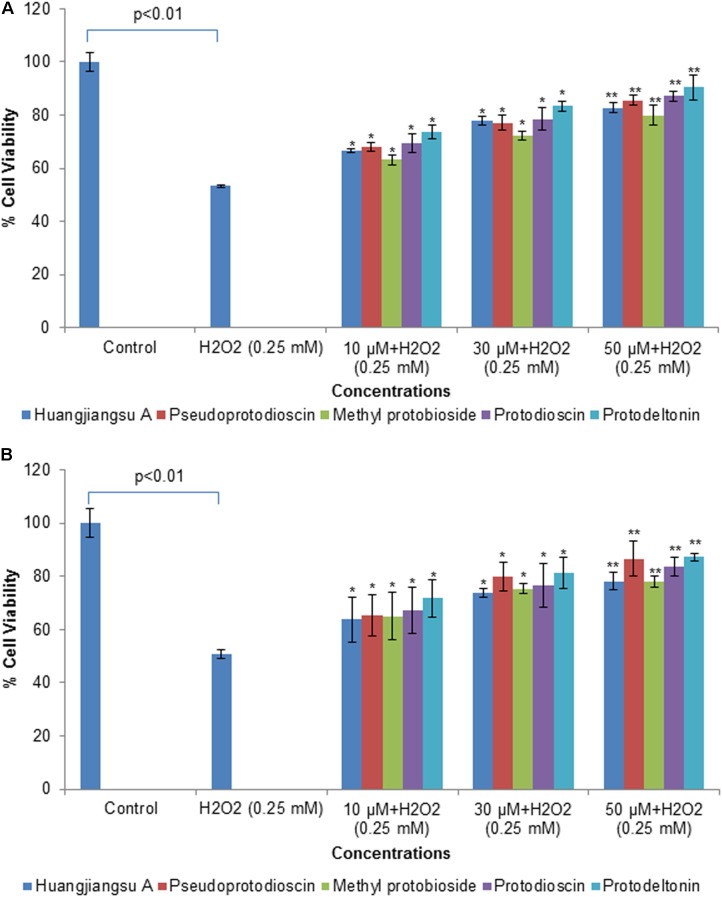
Assessment of protective potential of compounds on cell viability of HepG2 cells by **(A)** MTT assay and **(B)** NRU assay. Cells were exposed to 10, 30, and 50 μM of non-cytotoxic compounds for 24 h. Then the cells were exposed to H_2_O_2_ at 0.25 mM for 24 h. All values are given as mean ± SD. ^∗^*p* < 0.05, ^∗∗^*p* < 0.01 versus H_2_O_2_.

To illustrate cytoprotective effects of the non-cytotoxic compounds (huangjiangsu A, pseudoprotodioscin, methyl protobioside, protodioscin, and protodeltonin) on cell viability of HepG2 cells against cytotoxicity induced by the H_2_O_2_, the HepG2 cells were incubated with 10, 30, and 50 μM concentrations of test compounds for 24 h and then with cytotoxic concentration (0.25 mM) of H_2_O_2_. The H_2_O_2_-induced cell death and hepatoprotective effect were determined by MTT and NRU assays and morphological changes. The protective potential of each compound as observed in HepG2 cells is presented in **Figure 5**. A significant (*p* < 0.01) reduction in cell viability was observed in HepG2 cells following the exposure with H_2_O_2_ (0.25 mM) for 24 h by MTT assay (**Figure 5A**), and NRU assay (**Figure 5B**). However, a concentration-dependent increase in the cell viability was observed in HepG2 cells pre-exposed to huangjiangsu A, pseudoprotodioscin, methyl protobioside, protodioscin, and protodeltonin compounds at 10, 30, and 50 μM for 24 h. The increase in cell viability was observed to be 13, 24, and 29% by huangjiangsu A; 15, 24, and 32% by pseudoprotodioscin; 10, 19, and 26% by methyl protobioside; 16, 25, and 34% by protodioscin; and 20, 30, and 37% by protodeltonin at 10, 30, and 50 μM, respectively, in pre-exposed HepG2 cells (**Figure 5A**). Similar to MTT assay, a concentration-dependent protective effect was also observed in NRU assay. The increase in cell viability by NRU assay was observed to be 13, 23, and 28% by huangjiangsu A; 15, 30, and 36% by pseudoprotodioscin; 14, 25, and 28% by methyl protobioside; 17, 26, and 33% by protodioscin; and 21, 31, and 37% by protodeltonin at 10, 30, and 50 μM concentrations, respectively, in pre-exposed HepG2 cells (**Figure 5B**). The results of this study showed that pre-exposure of cells to the test compounds significantly increased the viability of HepG2 cells against H_2_O_2_-induced cell death. Our results support the previous studies that compounds isolated from natural products exhibited significant hepatoprotective effects against H_2_O_2_ (Xia et al., 2017) and against other toxicants such as acetaminophen and CCl_4_ (González et al., 2017). Several studies have also demonstrated that various compounds isolated from plants exhibited prominent cytoprotective potential against H_2_O_2_-induced cell death in different cell system (Yang et al., 2015b; Wang et al., 2015).

### Morphological Changes

**FIGURE 6:**
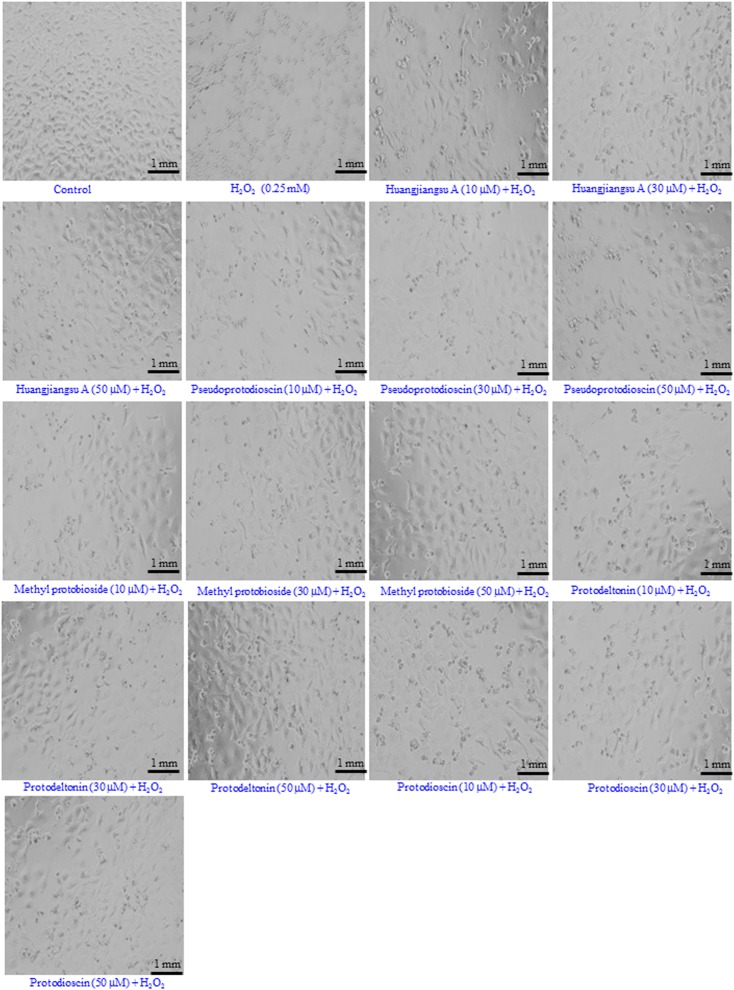
Morphological changes observed in HepG2 cells. Cells were pre-exposed with non-cytotoxic compounds for 24 h and then H_2_O_2_ (0.25 mM) for 24 h. Images were acquired using a phase contrast inverted microscope at 20× magnification. Each scale bar = 1 mm.

The morphological changes observed in HepG2 cells pre-exposed to compounds from *D. villosa* and then H_2_O_2_ for 24 h are summarized in **Figure 6**. As shown in the figure, H_2_O_2_ at 0.25 mM concentration induced characteristic morphological changes, such as cell shrinkage, appearance as rounded bodies and loss of adhesion capacity as compared to the control. However, HepG2 cells pre-exposed to 10, 30, and 50 μM concentrations of huangjiangsu A, pseudoprotodioscin, methyl protobioside, protodioscin, and protodeltonin restored their original morphology similar to the control in a concentration-dependent manner. The preservation of the normal morphology of HepG2 cells observed by phase contrast microscope indicated the preventive role of the compounds against H_2_O_2_-induced morphological damage.

### Protective Potential of Compounds Against H_2_O_2_ Reduced GSH Level

**FIGURE 7:**
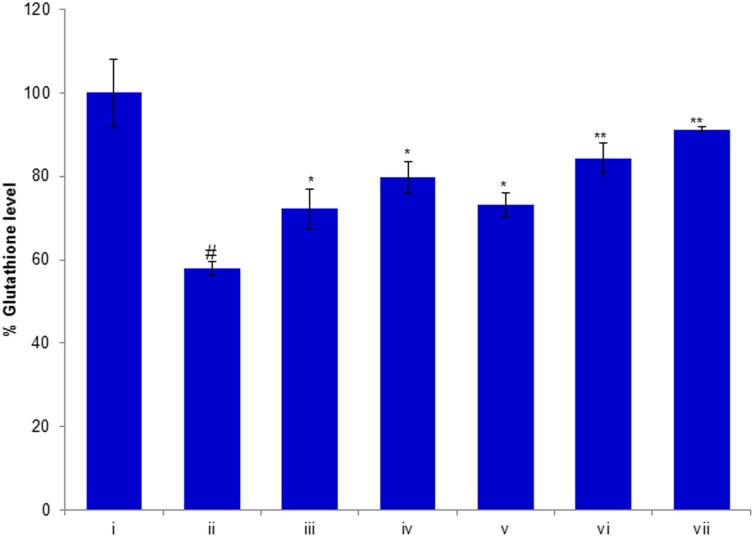
Protective potential of compounds isolated from *Dioscorea villosa* on depletion in glutathione level. HepG2 cells were exposed to 50 μM of non-cytotoxic compounds for 24 h prior to the addition of H_2_O_2_ for 24 h. All values are represented as mean ± SD. **(i)** Control; **(ii)** H_2_O_2_ (0.25 mM); **(iii)** Huangjiangsu A (50 μM) + H_2_O_2_ (0.25 mM); **(iv)** Pseudoprotodioscin (50 μM) + H_2_O_2_ (0.25 mM); **(v)** Methyl protobioside (50 μM) + H_2_O_2_ (0.25 mM); **(vi)** Protodioscin (50 μM) + H_2_O_2_ (0.25 mM); and **(vii)** Protodeltonin (50 μM) + H_2_O_2_ (0.25 mM). #*p* < 0.01 versus control, ^∗^*p* < 0.05 and ^∗∗^*p* < 0.01 versus H_2_O_2_ exposure.

In order to evaluate the effects of compounds of *D. villosa* on antioxidant defense systems, the glutathione (GSH) level was measured in H_2_O_2_ exposed HepG2 cells. The results of this study showed that GSH level in H_2_O_2_ exposed HepG2 cells was significantly decreased by 43% (*p* < 0.01) as compared to the control (**Figure 7**). However, as shown in the figure, pre-exposure of cells to huangjiangsu A, pseudoprotodioscin, methyl protobioside, protodioscin, and protodeltonin at 50 μM concentration significantly increased the GSH level in H_2_O_2_ exposed HepG2 cells. The increase in GSH level was 15% by huangjiangsu A, 22% by pseudoprotodioscin, 16% by methyl protobioside, 27% by protodioscin, and 34% by protodeltonin at 50 μM (**Figure 7**). Our results showed that pre-exposure of cells to the test compounds effectively restored the GSH level. It is well known that exogenous exposure of H_2_O_2_ can increase the intracellular ROS generation and induced cellular oxidative damage in hepatic cells and it can be eliminated by hepatocyte antioxidant defense mechanisms (Xia et al., 2017). GSH is a main non-enzymatic antioxidant, which plays an important role in the cellular defense system against oxidative stress (Scharf et al., 2003). It has been reported that glutathione peroxidase catalyses GSH oxidation to GSSG at the expense of H_2_O_2_ and glutathione reductase recycles oxidized GSH back to reduced GSH (Al-Sheddi et al., 2016). Thus, it could be concluded that the pre-exposure of HepG2 cells to these compounds quenches the intracellular destructive peroxide and increases the level of glutathione thus enhancing the antioxidant status and protecting the cells against the H_2_O_2_-induced damage.

### Protective Potential of Compounds Against H_2_O_2_-Induced ROS Generation

**FIGURE 8:**
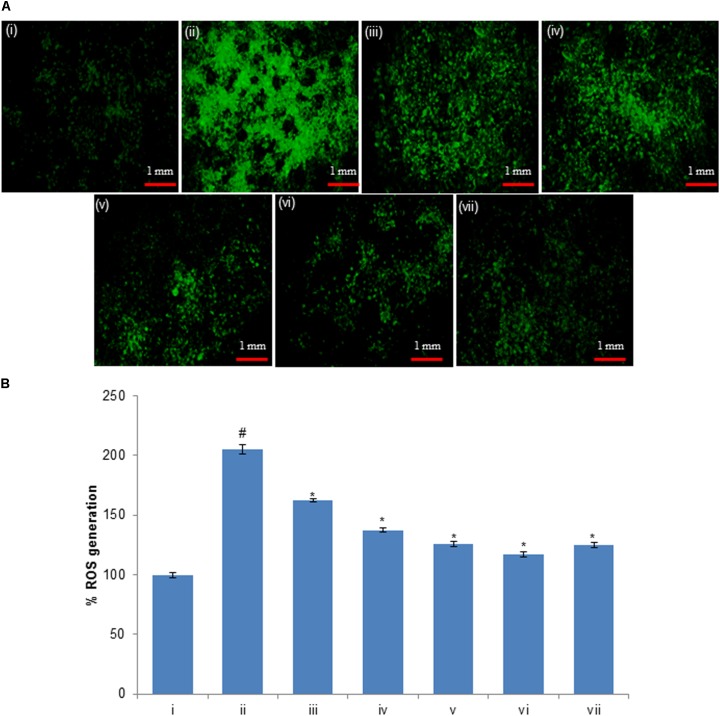
**(A)** H_2_O_2_-induced ROS generation and ameliorative effect of pre-treatment of compounds in HepG2 cells and **(B)** percent change in ROS generation. ROS generation was evaluated using dichlorodihydrofluorescein diacetate (DCFH-DA) dye. **(i)** Untreated control; **(ii)** H_2_O_2_ (0.25 mM); **(iii)** Huangjiangsu A (50 μM) + H_2_O_2_ (0.25 mM); **(iv)** Pseudoprotodioscin (50 μM) + H_2_O_2_ (0.25 mM); **(v)** Methyl protobioside (50 μM) + H_2_O_2_ (0.25 mM); **(vi)** Protodioscin (50 μM) + H_2_O_2_ (0.25 mM); and **(vii)** Protodeltonin (50 μM) + H_2_O_2_ (0.25 mM). ^#^*p* < 0.01 versus control, ^∗^*p* < 0.05 versus H_2_O_2_ exposure. Each scale bar = 1 mm.

The level of intracellular ROS generation in HepG2 cells was evaluated using a DCFH-DA fluorescent probe. The HepG2 cells were pre-exposed to 50 μM of compounds (huangjiangsu A, pseudoprotodioscin, methyl protobioside, protodioscin, and protodeltonin) for 24 h and then H_2_O_2_ for 24 h. The results showed that the DCF fluorescence intensity in H_2_O_2_-treated HepG2 cells was significantly increased to 104% as compared to the control group (**Figure 8**). However, the fluorescence intensity was notably reduced up to 42, 67, 79, 87, and 80% in HepG2 cells pre-exposed to 50 μM of huangjiangsu A, pseudoprotodioscin, methyl protobioside, protodeltonin, and protodioscin compounds, respectively (**Figure 8**). As shown in figure, compound huangjiangsu A showed the highest scavenging activity of intracellular ROS generation. It is reported that exogenous exposure of H_2_O_2_ resulted in accumulation of intracellular ROS generation, which plays an important role in cell differentiation, cell proliferation, and cell death (Park, 2013). ROS generation is well known to be associated with the principle of oxidative stress (Schieber and Chandel, 2014). Oxidative stress is the net result of an imbalance in the amount of ROS generation and destruction, which is involved in causation and consequences of many diseases (Aruoma, 1998; Rodriguez and Redman, 2005). It is also been reported that induction in ROS generation plays an important role in the hepatocellular damage (Jiang et al., 2014). Since H_2_O_2_-induced oxidative stress and ROS generation have been previously reported, we used H_2_O_2_ to induce ROS generation in HepG2 cells. In this study, an increase in the ROS generation indicated that H_2_O_2_ could cause oxidative stress in HepG2 cells. The decrease in the intracellular ROS generation in HepG2 pre-exposed to 50 μM showed the protective potential of various compounds against H_2_O_2_-induced ROS generation. Our results are also in accordance with the previous reports showing that the administration of compounds isolated from plant suppresses the level of ROS generation (Yang et al., 2015a).

## Conclusion

The present study demonstrated the cytotoxic and cyto-protective potential of selected steroidal glycosides, i.e., zingiberensis saponin I, dioscin, deltonin, progenin III, huangjiangsu A, pseudoprotodioscin, methyl protobioside, protodioscin, and protodeltonin, isolated from *D. villosa*. Furostane-type steroidal glycosides (huangjiangsu A, pseudoprotodioscin, methyl protobioside, protodioscin, and protodeltonin) were found to attenuate H_2_O_2_-induced cytotoxicity in HepG2 cells. Their protective effect seems to be mediated by inhibiting intracellular ROS generation and restoring the GSH level. Together, results from the present study suggest that the constituents of *D. villosa* can protect HepG2 cells against H_2_O_2_-induced oxidative damage through antioxidant activity.

## Author Contributions

MS and IK designed the experiments. ZA, AC, and MS performed the experiments. ZA and MS analyzed the data and drafted the manuscript. IK and AC supervised the study. All the authors read and approved for the publication.

## Conflict of Interest Statement

The authors declare that the research was conducted in the absence of any commercial or financial relationships that could be construed as a potential conflict of interest.
